# Determinants of technology adoption and continued use among cognitively impaired older adults: a qualitative study

**DOI:** 10.1186/s12877-022-03048-w

**Published:** 2022-04-28

**Authors:** Samantha Dequanter, Maaike Fobelets, Iris Steenhout, Marie-Pierre Gagnon, Anne Bourbonnais, Samira Rahimi, Ronald Buyl, Ellen Gorus

**Affiliations:** 1 Department of Public Health Sciences, Biostatistics and Medical Informatics (BISI) Research Group, Vrije Universiteit Brussel, Brussels, Belgium; 2 Department of Nursing Sciences, Université Laval, Quebec, Canada; 3 Faculty of Nursing, Université de Montreal, Montreal, Canada; 4 Department of Family Medicine, McGill University, Montreal, Canada; 5 Lady Davis Institute for Medical Research, Jewish General Hospital, Montreal, Canada; 6 MILA – Quebec Artificial Intelligence Institute, Montreal, Quebec, Canada; 7 Department of Gerontology, Faculty of Medicine and Pharmacy, Frailty in Ageing (FRIA) Research Group, Vrije Universiteit Brussel, Laarbeeklaan 103, 1090 Brussels, Belgium; 8 Department of Geriatrics, UZ Brussel, Laarbeeklaan 101, 1090 Brussels, Belgium

**Keywords:** Cognitive impairment, Mci, Dementia, Informal caregivers, Professional caregivers, Interviews, Focus group, Thematic analysis

## Abstract

**Background:**

Technology offers opportunities to support older adults with mild cognitive impairments to remain independent and socially connected, but is often not used. Although determinants of technology use among older adults in general are well studied, much less is known about how these factors impact technology use behaviour in cognitively impaired older adults. This study aimed to bridge this gap in research by examining the factors underlying technology use in community-dwelling older adults with mild cognitive impairments.

**Methods:**

We applied a generic qualitative design and used 16 semi-structured interviews to collect data from Belgian (Flemish) community-dwelling older adults diagnosed with Mild Cognitive Impairment or dementia and informal caregivers. To get data from different perspectives, a focus group with professional caregivers was added. We used thematic analysis with an inductive approach to identify and select themes from the data.

**Results:**

We identified two themes: introduction of technology and determinants of technology adoption and continued use. Successful technology adoption in cognitively impaired older adults is need-driven and subject to individual, technological and contextual characteristics. Specific for older adults with cognitive impairments are the importance of disease awareness and cognitive ability for adoption and continued use, respectively. Although social support can be a valuable alternative to technology, it is an important facilitator of continued technology use in these older adults. Similarly, integration of technologies in daily routines can buffer discontinuation of technologies.

**Conclusions:**

Future research is encouraged to validate our findings in a postpandemic era and to further develop a novel theoretical framework for technology acceptance among older adults with cognitive impairments. Moreover, identification of crucial determinants as well as strategies to remove use barriers are also important future research tasks. Clinical practice should focus on improving disease awareness to facilitate technology adoption and policies should invest in training and support of professional caregivers and in reimbursement strategies to facilitate implementation of technology in practice.

**Supplementary Information:**

The online version contains supplementary material available at 10.1186/s12877-022-03048-w.

## Background

Worldwide ageing is going fast and is not merely a story of prosperity as it also entails global challenges [1, 2]. Since age is the most important risk factor for neurocognitive disorders, one of those challenges is the increasing prevalence of neurocognitive decline caused by Mild Cognitive Impairment (MCI) and dementia [3]. These disorders result in functional limitations, threatening the potential to age autonomously at home without additional care [4–6]. A possible strategy to assist these community-dwelling cognitively impaired older adults involves the implementation of innovative technologies [7]. These technologies are sometimes referred to as e-Health technologies [8] or gerontechnologies [9] and can be applied to divergent need domains of older adult life. Among them are mobile apps, assistive technologies, monitoring or ambient assisted living technologies, exergames and telemedicine technologies for remote health counselling [10, 11]. Recent research has demonstrated beneficial effects of these technologies on a variety of health and well-being outcomes in older adults with and without cognitive impairments [10, 12–15].

However, to profit from these technologies, they need to be used by older adults. The past decade, research has revealed the so called grey digital divide, a phenomenon referring to the unequal access to and limited use of internet among older adults, as compared to younger adults [16, 17]. Extensive research relates sociodemographic characteristics to this phenomenon, including the female gender, living alone, living in an urban environment and lower education [18–21]. However, this can also be interpreted as a generational issue, as the oldest older adults often lack adequate previous workplace experience with technology, financial resources or social support and tend to present different consumer behaviour [20–22]. Research shows that poorer physical health [23–25], lower psychological well-being [25] and lower functional ability [25] are associated with lower technology use in older adults. Cognitive impairment has also been negatively related to technology use [20, 23, 26–29]. However, the reasons for this lower use behaviour in older persons with mild cognitive impairments (PMI) remain underexplored. Some sociodemographic (e.g. age, gender, education and living situation) and individual characteristics (e.g. better health and psychologic well-being) correlating to technology use in PMI are similar to those in the general older adult population [27]. Nevertheless, the question remains which determinants are specific to PMI.

Classical theories on technology acceptance in adults include important predictors such as perceived usefulness and ease of use [30–32], but lack essential predictors that are specific to the population of PMI, such as biophysical (e.g. the cognitive decline) and psychosocial (e.g. the need to remain independent or to feel safe) characteristics [33–35]. With regard to ease of use, complexity of technology certainly plays an obstructing role among PMI [36]. However, this can’t be the only explanation for low technology use in this subpopulation, as LaMonica et al. [29] found that the lower technology use in PMI was not the result of experiencing more difficulties with technology engagement. Possibly, unequal access, unsuccessful adoption or other user-related reasons (e.g. negative attitude or low self-efficacy) are at the basis of this phenomenon. However, research on this topic in the specific population of PMI is scarce [34, 37]. A recent qualitative study with technology-using PMI by Blok et al. [37] revealed that perceived usefulness is the most important predictor for technology acceptance in PMI, but is also specifically related to the satisfaction of social and emotional needs in this group. These needs include the desire to maintain control over life, to support relationships and to assist daily activities. Furthermore, familiarity and former experience with technology were reported to facilitate technology acceptance and use in PMI, whereas lack of personalization and lack of social support were obstructing factors for use [34, 36]. Lastly, technology use in older adults not only depends on sociodemographic or individual determinants, but also from the dynamic interplay of these determinants with the social network and the physical and organizational environment [38–41]. Therefore, implementation strategies, including promotion, policy changes and organizational collaboration, are important to implement technology use in a sustainable way.

To conclude, although there is strong evidence of less technology use by PMI [20, 23, 26–29], a strong evidence base for the reasons of use and non-use, specifically in PMI, is lacking. Moreover, the limited research in this specific population [34, 37] applied a theoretically driven data-analysis and did not thoroughly explore determinants of non-use of technology. Therefore, this study aimed to examine the factors underlying technology use as well as non-use in PMI by using an inductive generic qualitative design, including both technology using and non-using community-dwelling PMI, as well as informal caregivers (ICG) and professional caregivers (PCG).

## Methods

### Design

This study used a multi-source generic qualitative design [42] to collect data on the perceived facilitators and barriers towards using technology among community-dwelling PMI. This generic approach includes an inductive, data-driven analytical process in which no predefined theoretical frameworks are applied. Data were collected through semi-structured interviews with PMI and ICG and through a focus group of PCG.

### Participants and recruitment

All interviews were conducted between November 2018 and March 2020 and took place in the home environment of the PMI or at the geriatrics department of the University Hospital (UZ Brussel). The focus group was conducted in October 2019 at the UZ Brussel.

#### PMI and ICG

The study population included Dutch-speaking community-dwelling PMI aged 60 and over who had been formally diagnosed with MCI or mild dementia. Exceptions to the age criterion were allowed in case of young dementia. Participants with acute illness, severe auditory impairments or verbal communication disorders were excluded. All participants lived in the Flanders region (Dutch speaking part) of Belgium. Moreover, to obtain data from different perspectives, ICG were also invited to participate in this study. They had to be at least 18 years old and had to be actively involved in the care of the PMI as a primary caregiver. The PMI were given the choice to conduct the interview individually or in the presence of the ICG. To attract PMI with experience in technology use as well as PMI with no experience, purposive sampling was used. Multiple recruitment strategies were used. First, participants were recruited in the geriatrics and neurology departments of the UZ Brussel. Second, participants were recruited by the Flanders’ Centre of Expertise on Dementia (Expertisecentrum Dementie Vlaanderen) who informed potential participants through their digital newsletters and their social media channels.

#### PCG

PCG experienced in the care or support of community-dwelling PMI were purposively sampled for a focus group. These PCG consisted of in-hospital staff, including at least a medical doctor and multidisciplinary caregivers, as well as home care professionals (general practitioner, nurse and occupational therapist) and PCG of health insurance funds. This was realized by inviting staff members of the memory clinic of the UZ Brussel and PCG from their professional network. Additional recruitment was conducted through the networks of the staff members of the geriatric day clinic and of the researchers involved in the study.

### Data collection procedure

Ethical approval of this study was obtained by the Medical Ethics Committee of the UZ Brussel and Vrije Universiteit Brussel (B.U.N. 143201835242). All data were collected by a gerontopsychologist in full accordance with the guidelines for conducting research with PMI [43, 44]. At the start of the data collection, participants were asked to read and sign an informed consent form to demonstrate voluntary participation and consent to the audio-recording of the data. In case of a PMI with dementia, the ICG or legal representative was asked to additionally consent for the PMI’s participation. Main sociodemographic data, such as age, gender, profession (in the case of the focus group) or relatedness to the PMI (in the case of interviews with ICG) were collected through a short written survey. A semi-structured topic guide including open questions was developed based on discussion between all members of the research projects’ steering committee.

As an icebreaker, interviewed participants were first asked about their daily activities and experienced difficulties. Then they were asked whether they used or have used technologies and in case of a positive response, the relevant technologies were inventoried. Moreover, participants were asked for experienced facilitators and barriers to using technology. To generate and deepen discussion among PMI lacking experience with technology, photo-elicitation was added to the interviews [45]. Therefore, examples of technologies organized per application category (i.e. fall detection, medication management, communication, orientation and navigation, leisure) were presented to the participants, who were then asked to give their opinion on these technologies as well as the potential facilitators/barriers towards using them.

For the focus group, more active techniques combining brain storms and conceptual mapping were introduced. Thereby, PCG had to generate facilitators and barriers to using technology in PMI, write them down on scribble paper and sort them into the predefined categories Facilitators and Barriers. Before ending the focus group and interviews, participants were given a summary of the collected data by the interviewer, to allow them to confirm the main findings and to supplement with more information if needed. Participants were asked to contact the interviewer if any new ideas or perceptions regarding the research topic emerged after the appointment. An overview of the interview and focus group guide is presented in the Supplementary Information.

### Data analysis

The study took an inductive generic approach to data-analysis [42]. By targeting different participant groups (data triangulation), using different data collection methods (methodological triangulation) and conducting the data-analysis independently by at least two researchers, methodological quality was optimized [46]. Throughout the data analysis process, the qualitative analysis software package NVivo 12 was used [47]. First, the auditory data collected from the interviews and focus group were transcribed ad verbatim and, together with the field notes, thoughtfully and actively read by the interviewer. Thereby, memos including emerging reflections were written down by the interviewer to enhance data exploration. Meaningful data extracts from the transcripts were independently identified and coded at semantic level by the principal researcher (SD) and a Master student (DK) and regularly briefed and debriefed with other researchers (EG, MF) following a thematic data-analysis strategy [48]. Following this open coding process, similar codes were collated to form substantively related themes and subthemes. Thereby, labels were applied to differentiate codes describing factors related to adoption processes from codes describing processes of continued use. Adoption refers to the first contact with and new usage of technology, whereas continued use refers to long-term use behaviour of technologies that are already adopted. This categorisation process allowed for structuration of data into pre- and post-implementation stage determinants. The identified (sub)themes were then discussed with a third and fourth researcher (EG, MF) with experience in qualitative research, to redefine them and to form a meaningful framework from the data. Multiple consecutive thematic maps were constructed during the data analysis, as coding, identification and redefinition of themes were conducted in a recursive, non-linear manner. Since the relationships between the various codes and themes were clear and no new relevant information could be identified from the data of the interviews, data was considered saturated and no additional interviews were collected after March 2020.

## Results

### Participant characteristics

A total of 16 interviews were conducted: 7 duo-interviews (persons with mild dementia and their ICG), 1 interview with a person with dementia who did not have an ICG, 1 interview with an ICG (person with dementia had progressed to a more advanced stage of the disease by the time of the interview) and 7 interviews with persons with MCI. We also conducted a focus group consisting of 8 PCG (2 men, 6 women; mean age: 38.0 years; age range: 23–54 years) of different profiles: neurologist, geriatrician, general practitioner, 2 occupational therapists, social worker, home care service manager and home care nurse. The interviews lasted on average 1 h and 3 min and the focus group lasted 1 h and 51 min.

Table 1 denotes the sociodemographic and technology use related characteristics of the interviewed PMI as well as a brief overview of involved ICG. All but two PMI (with a diagnosis of young dementia) were older than 65 (mean age: 76.0 years; age range: 56–91 years) and 11 PMI had at least some experience with technology. In line with the respective clinical concepts, older adults with MCI experienced fewer functional limitations than older adults with dementia. Computer and smartphone use was much more frequent in the MCI group (71% and 57% respectively) than in the dementia group (25% and 13% respectively), as opposed to tablet use (43% in MCI group and 50% in dementia group) for which no apparent group differences existed. Tech support was often provided by children and grandchildren. The majority of ICG were spouses and were women (mean age: 60.0 years; age range: 50–75 years).

**Table 1:** Sociodemographic characteristics of the interviewed persons with mild impairment and informal caregivers

PA No	Group^a^	Gender	Age	ICG (Yes/No, gender, age, relatedness)	Marital status	Education level	Former professional occupation	Digitally literated	Digital appliances	Digital activities	Tech support of (grand)children
1	MCI	M	70	/	Married	HE	Interpreter & secretary	Yes	PC, mobile phone, TV	PC banking, reading newspaper, email^b^	/
2	MCI	F	90	/	Widow	LSE	Secretary	No	PAS, TV	/	/
3	MCI	M	77	/	Married	LSE + HNDE	Head of workshop and administration	Yes	PC, smartphone, tablet, TV, car GPS	Playing card games, email, WhatsApp, online banking, online tax administration, making online medical appointments, GPS navigation	Daughter
4	MCI	M	87	/	Married	LSE	Civil servant	No	Mobile phone, TV	/	/
5	MCI	M	83	/	Widower	LSE	Joiner	Yes	PC, smartphone, TV, car GPS,	Playing card games, watching TV, email, Facebook, Messenger, internet searches, agenda app, GPS navigation	Grandson
6	MCI	M	91	/	Widower	LSE	Civil servant	Yes	PC, tablet, smartphone, PAS	Skype, email, playing PC puzzle games, internet searches	Son, Granddaughter
7	MCI	M	76	/	Married	HSE	Commercial engineer	Yes	PC, smartphone, tablet, TV	Online banking, email, WhatsApp, FindMyCar, Google Maps navigation, mobile calendar	Grandchildren
8	DEM	F	75	Yes, F, 51, daughter	Widow	LSE	Secretary & housewife	No	TV, mobile phone, PAS	Playing puzzle games	Daughter
9	DEM	F	76	Yes, M, 75, spouse	Married	LSE + HNDE	Administrative assistant	Yes	PC, TV, calendar clock	Playing card games	Spouse, daughter
10	DEM	M	79	No	Divorced	LSE	Independent food salesman	No	mobile phone, TV	/	/
11	DEM	M	62	Yes, F, 62, spouse	Married	HSE	Offset printing technician for 20y; helpdesk technician	Yes	Tablet, mobile tracking application, tracking keychain	Playing puzzle games	Spouse, daughter
12	DEM	F	86	Yes, F, 60, daughter	Widow	HE	Teacher & housewife	Yes	mobile phone, tablet, calendar clock	Browsing, Skype^b^	Daughter
13	DEM	M	67	Yes, F, 63, spouse	Married	HE	Prevention advisor & energy consultant in educational services	Yes	mobile phone, tablet	Listening to music and reading the newspaper	Daughter
14	DEM	M	56	Yes, F, 55, spouse	Married	HE	IT-specialist	Yes	PC, TV, tablet, smartphone	Playing games on PC and smartphone	/
15	DEM	F	76	Yes, F, 50, daughter	Widow	HE	Teacher English-French in secondary education	No	TV, mobile phone, robotic lawnmower, PAS	/	/
16	ICG^c^	F	67	/	Married (spouse)	/	/	/	/	/	/

*PA No.* participant number, *OA* older adult, *HE* higher education (college or university), *LSE* lower secondary education (until age of 15–16 years), *HSE* higher secondary education (until age of 18 years), *HNDE* higher non-degree education (tertiary education distinct from higher education), *PAS* personal alarm system

^a^Group: MCI = participant with a diagnosis of MCI, DEM = participant with a diagnosis of dementia, ICG = informal caregiver

^b^Activities that are no longer executed, but have been in the past

^c^Solo interview with an ICG (spouse of a person with dementia)

### Identified themes

**Fig. 1:**
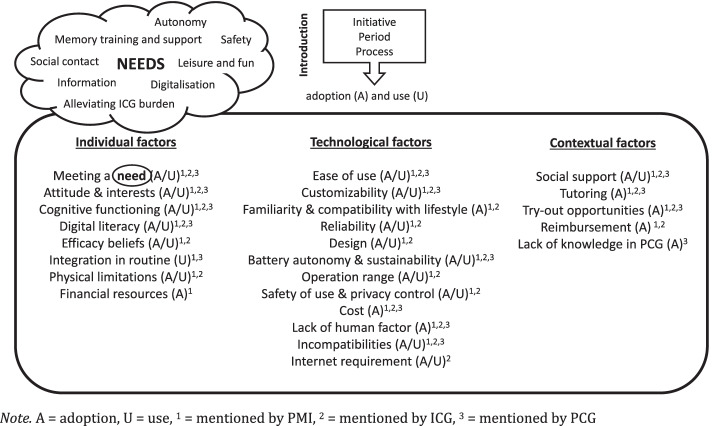
Identified themes and subthemes

From the interviews and focus group we identified two themes: introduction of technology and a major theme, i.e. determinants of technology adoption and continued use with three related subthemes. These themes and subthemes are summarized in Fig. 1.

#### Introduction of technology

Before technology is used, it needs to be introduced to an individual. It was clear from the interviews that this introduction process consisted of several aspects, including the period of introduction and the initiator(s) of technology use. Several key moments for introduction were identified: former professional life, retirement, triggering incidents (e.g. fall incidents), but also the moment of diagnosis of cognitive impairment.

*“(…) and I did, indeed, quite quickly start with it [mobile brain training games] and was like “That diagnosis is here. I have to make that work here!” [points at his head] You have to make that head work.” (PMI)*

The initiative for using and adopting technology could be taken by the older adult, family members (e.g. (grand)children gifting them), ICG (e.g. to support the care relationship) or PCG (e.g. to improve the PMI’s health or well-being).

*“(…)[Is shown a picture of an exergame-like stationary bike] Yeah, I know this application. I know it very well, because my physiotherapist already said: “Wouldn't you try using that too? Buy an exercise bike with which you can ride up hills and ride together with others.” (PMI)*

Furthermore, ICG and PCG stated that this introduction process is ideally a thoughtful process, which takes time and needs to be experienced as voluntary by the PMI.

*“(…) that takes time. If you want to introduce things, I notice with her [referring to the PMI with dementia]: that has to be done in phases. And you shouldn't force them because then (…) a reverse effect. So I don’t do that.”(ICG)*

However, not all participants had been introduced to technology. For these participants, the process of adoption and use of technology has not started (yet).

#### Determinants of technology adoption and use in PMI

A main theme concerned the determinants for technology adoption and continued use of technology by PMI (Fig. 1). Facilitators are those factors positively impacting the likelihood of adoption and continued use, whereas barriers refer to factors that obstruct the adoption and use in PMI. However, often factors can be a facilitator (e.g. digital literacy if it is high) and a barrier (e.g. digital literacy if it is low) at the same time. Individual, technological and contextual factors were identified.

##### Individual factors

These factors are related to the PMI’s and ICG’s personal characteristics.

Meeting a need

Participants reported that technologies need to be useful and personally relevant to a present or future need in order for them to adopt and use them (PMI, ICG, PCG). Several needs were identified, including memory training to prevent further cognitive deterioration (e.g. with puzzles and games) and support of daily structure (e.g. with calendar clocks) and activities (e.g. with agendas or medication management systems with reminders). Personal safety was another important need, comprising worries about emergency incidents (e.g. fall incidents, house fires, etc.) or complications with medication compliance (e.g. over- or underdosing) for which detecting and signaling technologies are found useful. Technology also plays a role in keeping contact with friends and family members as well as in making new social contacts (e.g. with email, messaging and social media apps, videoconference software, etc.) It is also a source of leisure (e.g. playing games or digitally looking at photos to pass time) and information (e.g. about the weather, news, purchasing articles, etc., for PMI and about dementia for ICG). Some PMI use the internet to digitalize administrative tasks (e.g. banking or tax filing) or see its potential as an alternative for physical appointments (e.g. to the general practitioner). Also, ICG believe technology can unburden them to a certain degree by supporting PMI in daily activities.

Highly discussed was the way in which technologies impact the need for autonomy and independence in PMI (PMI, ICG, PCG). Technology adoption can preserve independence, minimizing patronization of PMI by ICG or PCG and facilitate ageing in place in PMI by supporting them in activities of daily living. However, adopting new technologies was also perceived as potentially threatening for the sense of autonomy, resulting in PMI’s decision to delay the adoption of supportive technologies. These threats include the fear of limiting freedom of choice (e.g. to take medication whenever preferred) or the fear of no longer having to think for themselves and even be outsmarted by technology (PMI, ICG). Therefore, participants clearly preferred technologies adapted to the functional abilities of the PMI and focused on preserving self-sustainability, instead of invasive technologies that focused on taking over activities and responsibilities, as perceived in some robot or tracking technologies. To conclude, technologies are welcomed as long as they are supporting the PMI’ sense of autonomy, control and personal freedom.

*“This technology [a mobile GPS app for navigation] is more active than this one [a location tracking watch], which is more passive. Therefore, we would chose the active one as we want to maintain control of our situation and to be less dependent. (…) Because by wearing this [the watch] you know that others will always be able to find you, but what I wish is to find my way back home myself, for as long as I still can.” (PMI).*

In the absence of such needs PMI feel they have little motivation to adopt and use technology. This was demonstrated by PMI claiming that their functional capacities have not declined yet. Often these PMI attribute the need for such technologies to others who have worse functional abilities or who live alone. Other barriers to technology adoption are being satisfied with the current activity pattern or the availability of a good alternative to technology. The latter included anticipation strategies to prevent the cognitive impairment from interfering with independence (e.g. writing notes or enhancing the medication intake routine), as well as the presence of an ICG.

*“(…) But the problem has actually resolved itself because (…) She'll do that herself. She lays the pill on a serving tray near her bed and so she takes the pill right before goes to sleep. And that is really just a fixed moment that is clear to her.” (ICG)*

Attitude and interests

Some PMI and ICG considered technology as a symbol of scientific progress and innovation. They were motivated and open to learning new technologies. However, others had a more negative or even cynical attitude towards technology, overall lacking interest in technology, feeling too old to learn new technologies and doubting the extent to which technology could really support them. Moreover, some participants were concerned that technology would eventually replace or negatively impact all regular social contact (PMI, ICG, PCG).

*“(…) I think it’s sad. If they [grandchildren] keep busy with that all the time, then they see nothing of the things… around them. And then there is less chatter. (…) If one of my grandchildren would come to my house and would continuously be keeping busy with that little machine, I wouldn’t like that.” (PMI)*

The PMI’s attitude towards protection of shared information and disease awareness are two factors believed to influence the attitude on technology use.

*“My experience is that people who realize that they are forgetting and get frightened by this tend to cling to things that give them support and help, and that something like that is very welcome for them. In contrast to people lacking disease awareness.” (PCG)*

Furthermore, not only the attitude of the PMI, but also that of their environment is estimated essential for technology adoption among PMI (ICG).

*“There are certain thing that the person with dementia really likes but that the family doesn’t want because of how it looks for the outside world, a certain taboo atmosphere. For instance, seeing your mother with a doll is impossible, even though she is so happy with it and if it is the only way to calm her down.” (ICG)*

This also goes for PCG, as PMI estimated that some PCG are potentially not ready or open to use technologies (e.g. tele-conferencing) in their daily practice.

Cognitive functioning

The increased care needs resulting from the cognitive impairment in PMI can facilitate the introduction and adoption of technology. However, cognitive deterioration can also obstruct the adoption or continued use of technology in this group (PMI, ICG, PCG). This results from the difficulties in learning to use new technologies (adoption), as well as forgetting to use them or the deterioration of skills to use them (continued use). The latter includes impairments in the capacity to adapt to environmental changes.

*“So usually, I keep track of certain messages I receive, or photos, and I do this in a chronological manner. But, without my knowing about it, my wife moved my icons [on the computer desktop] and relocated them per subject. And now, it’s a disaster. (…) I feel like “Whatever, I don't want to look at it anymore.”” (PMI)*

Digital literacy

The degree of experience with technology is perceived as important for technology adoption and use (PMI, ICG, PCG). It can serve as a facilitator in PMI who have learned to use technology in their spare time or in a former working environment. In contrast, lacking former experience with technology was mentioned as a clear barrier to adoption of new technologies and to more extensive use of technologies that were already in use.

Moreover, digital literacy was perceived as a cohort related aspect, as illustrated by a partner (ICG) of a PMI diagnosed with advanced dementia.

*“(…) But the current group of people with dementia, the older people, were not raised with electronics. They lived in the days in which there was no telephone in the house at all. (…) It's a very different generation from yours.” (ICG)*

Efficacy beliefs

The beliefs PMI (self-efficacy beliefs) and ICG (other-efficacy beliefs) had about the PMI’s ability to learn and use new technologies were related to adoption and use. These include beliefs about knowledge, skills and degree of control one has with regard to unfamiliar technologies. Positive efficacy beliefs were reflected in the perception that PMI can learn new technologies, despite advanced aged or cognitive impairment. Negative efficacy beliefs reflected few expectations about the PMI’s ability to adopt new technologies. PMI and ICG related these negative presumptions to the PMI’s digital literacy level as well as to the complexity level of the technology.

*“Even if you would give me this for free [a GPS], I’d still don’t want to have it. (…) No, I know I’m going to struggle with it… And you need to install it and… No.” (PMI)*

In some PMI, these negative beliefs were associated to feelings of shame about not being able to use new technologies or about giving up the use of technologies that they don’t master anymore.

Integration in routine

For continued use of technology by PMI, integration in daily routine was important (PMI, ICG), this could improve preservation of skills. If technology use is not integrated in daily routine, the use of it will likely be discontinued (PMI, ICG).

Physical limitations

Certain aspects related to physical decline in PMI were identified as barriers to adoption and continuous use of technology (PMI, ICG). These included visual impairments (e.g. with small displayed text), impaired finger or grip strength or pain due to degenerative joint diseases (e.g. arthrosis) affecting the hands, making it impossible to manipulate keys or buttons.

*“Even if older adults can no longer speak, they may still be able to do things on a tablet. So I think the accessibility and customizability of the technology is something that is progressing in a very positive way. Icons on a screen, the size they are, the color they have, the contrast,…. For people with macular degeneration or so, there are many possibilities.” (PCG)*

Financial resources

Adoption of technology is also depending on the PMI’s financial resources. PMI often mentioned having a relatively low retirement income that limits their opportunities to purchase new technologies, certainly if these are highly priced.

##### Technological factors

A second category of factors are those that are associated to characteristics of technologies themselves.

Ease of use

Technology should preferably be easy to handle and require little thought or active effort of the PMI, e.g. cell phones specifically designed for seniors. Technology that is perceived as too complicated or too technical is therefore at risk of being rejected or discontinued by PMI. Hence, technologies that require little interaction and no peripherals (e.g. keyboard, mouse, etc.), as is the case in tablet computers (PMI, ICG), are preferred (PMI, ICG). These also consist of detecting and signalling technologies which are especially valuable because they don’t require a learning curve and cannot be forgotten to be used at crucial moments. These were preferred specifically for low to non-digitally literate PMI and PMI with advanced cognitive impairment. In line with this, interest in voice-controlled technologies was identified.

*“Television use, for example. All those buttons [on the remote control] (…) For a lot of people that's (…) Why doesn’t there exist a TV which you can ask “TV, turn on! TV, go to channel one!” That would be just fantastic. (…) But there are a lot of people who just don't know what to do with it. All they can do is to turn on and turn off the TV and then they start searching on how to operate it. Imagine that you can say all those commands to the television set, wouldn’t that be great?” (PMI)*

Lastly, one PMI reported preferring to use his personal computer for writing letters or emails, due to its keyboard that facilitates an easy user experience.

Customizability

Technologies should be adjusted to the PMI’s personal preferences, cognitive capacities and physical limitations (PMI, ICG, PCG). All participant groups mentioned that personalization of technologies (e.g. prompting with personal name, voice commands voiced by the ICG,…) can generate a more personal and familiar user experience, and thus improve adoption and continued use, specifically in robotics systems and assistive technologies. Moreover, with progressing cognitive impairment, technologies should be able to change from more complex and challenging to more intuitive and easy to use (PMI, ICG, PCG). The option to set a difficulty level when playing a memory training game or the option to choose from different types of medication management systems depending on the cognitive support needs, are just a few examples.

*“There are games that are very (…) I mean, with cubes for instance. I've already come across these, but I’d rather play games with words for which you really need to use your brain. It can’t be too easy. (…) I shouldn't be too condescending about that but we [PMI] like to be challenged intellectually too, you know.” (PMI)*

Familiarity and compatibility with lifestyle

Specifically for adoption, technologies that feel familiar and are compatible with the current lifestyle are preferred (PMI, ICG). A recurring example is the use of a smartwatch prompting notifications to support medication management. According to PMI, such smartwatches should also be functional and look like a normal watch, in order to integrate its use in daily routine. Another example is the adoption of smart monitoring or tracking keychains in PMI that already frequently have their keys on them.

*“I have a preference for the key chain [for tracking] over the watch because we currently don’t have the habit to wear a watch. And that key chain, I think it’s the best solution, because then I can just hang it next to my keys.” (PMI)*

Furthermore, technologies that cannot flexibly adapt to personal routines are also at risk of not getting adopted by PMI. For instance, one PMI remarked that a medication dispenser should be able to flexibly respond to his being away on daytrips, otherwise it would not fit its current lifestyle and be worthless to him. Therefore, mobility and wearability of certain technologies is considered important (PMI, ICG, PCG). By wearing wireless and portable technologies such as smartwatches for fall detection, daily activity prompting or medication reminding, PMI can continuously and at multiple locations benefit from their functions. In addition, puzzle games have an added value compared to traditional paper–based puzzles, as they too are always within reach of the PMI.

Reliability

Although it seems evident, PMI and ICG remarked that technology needs to work as it should be working, and when it needs to. They want to be able to rely on technology, specifically in the context of medication management support, fall detection and location tracking. One ICG emphasized that this factor is even more of importance in the adoption of costly technologies.

Design

Another frequently mentioned factor, specifically applied to wearable technology, concerned aesthetics and wearing comfort (PMI, ICG). Supportive smartwatches should be elegant and fun to wear in order for PMI to be willing to adopt them. They should not be too big and should not attract too much attention. Furthermore, wearing comfort seems to be of high importance, as participants mentioned incidents in which uncomfortable smartwatches for fall detection were taken off at bedtime, resulting in an increased risk of undetected fall incidents at night-time. From this example, it is clear that design plays an important role in adoption, but also in sustained and proper use of certain supportive technologies.

Battery autonomy and sustainability

A highly reported concern is that of the battery autonomy of technological appliances (PMI, ICG, PCG). This involves worrying that appliances will stop working when they run out of battery capacity or in case of power failure, making it impossible for the PMI to use them properly. ICG frequently expressed concerns involving the timely charging of appliances as PMI sometimes tend to forget to charge their appliances or fail to retrieve or correctly apply charging cables.

Hence, as do PMI, they have a preference for technological appliances that have long battery autonomy and therefore require less charges. One PMI actively anticipated the battery draining of his technological appliances.

*“I keep an eye on that, and regularly charge the iPad and the iPhone. (…) I do this almost every night: before I go to sleep I look at my appliances and check whether I have to charge them yet, and then I decide “I still don't have to charge it” or “Yes, I will charge it during the night, while I am asleep.”” (PMI)*

Furthermore, an ICG also mentioned considering aspects of sustainability and eco-friendliness when searching for supportive technologies for the PMI. This was illustrated by a clear preference for batteries that are not too easily worn-out and that are recyclable.

Operation range

PMI and ICG also remarked that the functional range of an appliance should be sufficiently large, as this could otherwise result in rejection or limited use of the appliance. This factor is mainly discussed in the context of technologies for location tracking or medication management for PMI who frequently go outside. As these appliances are often stationary they are only useful when the PMI is located near the appliances. Thus, technologies that function beyond these boundaries have added value and are highly welcomed.

Safety of use and privacy control

Safety of use is another issue mentioned by PMI and ICG. This refers to concerns about trustworthiness of information on the internet as well as issues of internet and privacy security. PMI report sometimes feeling insecure when encountering cumbersome pop-ups or software updates as they fear these contain safety hazards. A few PMI also mentioned being worried that banking apps will one day be subject to hacking and theft. In contrast, one PMI explicitly stated blindly trusting banking apps as he believed that they are among the safest apps. To counter these safety related insecurities, PMI often turn to family members, mainly (grand)children, who clarify whether the PMI is exposed to real risk or not.

Another issue is the violation of the PMI’s personal privacy by continuous tracking of their whereabouts (PCG). One way to tackle this issue is by letting the PMI chose with whom data is shared (PCG). This would allow for tracking permissions that selectively register and share data when the PMI actively pushes a button or at all time, if the PMI choses so. With this argument, PCG emphasize the importance of empowerment of PMI.

*“What I also forgot to say is that I really see an evolution in camera surveillance. I currently work with a lot of children of PMI who place cameras everywhere, and then I ask them: “Does your father or mother agree to this?” (PCG)*

Cost

A barrier frequently reported was that of high purchasing costs of technological solutions (PMI, ICG, PCG). Many participants assess these costs when considering adoption of technologies, but often find technologies too expensive for PMI’s budgets which results in PMI not buying them. One PMI mentioned anticipating overspending by comparing the purchasing costs of technologies on the internet.

Lack of human factor

Specific for adoption processes is the human factor (PMI, ICG, PCG). This refers to characteristics of technologies that facilitate a regular humanlike experience and satisfy PMI’s needs for familiarity, proximity and tactile contact. As robotic companions or supportive devices currently lack humanlike interaction, movement and voicing capacities, this factor is currently mainly evaluated as a barrier for adoption. Therefore, robotic companions are currently not considered a valuable alternative for home care staff or personal contacts by all participants.

*“I’d rather stick with my home care nurse than use such a robot (…) As long as I have the luxury of asking someone “Good morning. How was the traffic today? And do you’ve got other clients to take care of?”, so the little things… This way, you still meet other people” (PMI)*

Incompatibilities

When faced with the decision to adopt new technologies, PMI and ICG also consider the compatibility of these technologies with the ones already owned. For instance, a PMI lost interest in a visualization-driven stationary bike after learning that his own television monitor could not be connected to it. This opinion was shared by an ICG who stated that technologies should be able to connect to the ones already owned, preventing unnecessary costs. Furthermore, compatibility of technologies (e.g. applications) with the internet network a PMI is on, as well as the native language of the PMI using them was mentioned as being crucial for adoption.

Internet requirement

Lastly, the requirement of having an internet connection to use a certain technology among PMI currently not having an internet connection, is also a barrier (ICG).

##### Contextual factors

The third category of factors refer to the social and organisational environment surrounding the first (individual) and second (technological) category of determinants.

Social support

Besides introducing technologies, members of the social network of a PMI, such as (grand)children, can advise the PMI on which technologies to adopt or help them with specific questions concerning use safety. Furthermore, they serve as a safety net who the PMI can turn to for assistance. This includes supporting PMI in the process of learning to use a new technology, as well as helping them when they encounter technical issues or difficulties due to increasing cognitive impairment. In the latter case, ICG remind PMI to use technology to counter symptoms of disinterest or apathy, or help them to overcome obstacles when using technology. The lack of social support was reported as a barrier for both adoption and continued use of technology among PMI.

Furthermore, the presence of a social support network can also serve as an alternative to technology adoption. This was the case for PMI who felt that their ICG could take care of most of their current or future needs.

*“As long as it is the two of us, I wouldn’t necessary need it. But if I had to end up alone, then I might possibly consider it.” (PMI)*

Tutoring

Besides the help of the social support network or ICG, PMI can be educated to use new technologies by others (PMI, ICG, PCG). Professional caregivers, such as family care assistants, can initiate or follow-up this process, provided that they have enough time to do this (ICG, PCG). Next to PCG, PMI can learn to use new technologies in senior technology classes or, as one PMI preferred, with the help of the seller of the device.

Try-out opportunities

Another important factor for adoption of new technologies was the opportunity to try them out before having to purchase them (PMI, ICG, PCG). Participants reported often not being able to test a technology, resulting in difficulties for PMI’s to form an opinion on the technology and in reluctance towards purchasing it.

Reimbursement

As discussed, the combination of limited financial resources and highly-priced technology often results in rejection of their adoption. PMI and ICG believe that governmental action, for instance by (partial) reimbursement of a broad range of technologies could help to remove this barrier, resulting in greater accessibility for all PMI. However, as one dyad of PMI and ICG remarked that reimbursement of personal alarm systems is administratively still very complex, attention should also be focused on simplifying these procedures.

Lack of knowledge in PCG

Besides PMI, PCG also experience a lack knowledge of existing technologies and the resources to find supportive technological solutions. Therefore, they don’t feel confident to recommend them to PMI, which obstructs the implementation of technologies in practice.

## Discussion

### Principal findings

In this qualitative study, we aimed to get a comprehensive understanding of the determinants of technology adoption and use in community-dwelling PMI. We included data from different perspectives (i.e. different participant groups) and applied different data collection methods. The inductive data-driven analysis resulted in the unprejudiced identification of themes from the data. We compared our results with previous research and draw four main conclusions.

First, our data suggest that adoption and use processes are not completely the same. This distinction is in line with earlier research, but is often overlooked in studies on acceptance of technology among older adults, with the majority of these studies only reporting on the pre-implementation stage [49]. Factors that are known to specifically influence adoption, such as general attitude, efficacy beliefs and cost [49] were also identified in our study. However, our study results also show great overlap of adoption and use processes, suggesting that a lot of the determinants continue to influence use behaviour far past technology adoption. Moreover, factors (e.g. concerns about reliability, forgetting to use or losing technology and experiencing limitations in the operation range) that were linked to the post-implementation stage in previous research were also mentioned by PMI who did not had experience with technology yet. Possibly, the photo-elicitation technique inspired these PMI to envision these scenarios. However, this shows that most of these factors are considered as early as in the pre-implementation stage. Therefore, our results confirm recent research findings pointing out the dynamics and interplay of these factors [38–41].

Second, introduction of technology precedes adoption and use. According to our results, adoption can be the result of a PMI’s own initiative, but is also often initiated by ICG or PCG. This finding follows previous research [34, 37]. Thereby, ICG explicitly stated that PMI need to experience the adoption process as unrushed and voluntary. This was also emphasized in the work of Blok et al. [37] who argumented that perception of control is particularly relevant for adoption and continued use of technology by PMI. Besides in the (former) workplace environment, introduction of technology to PMI is often triggered by key moments (e.g. retirement, safety incidents or the moment of diagnosis of cognitive impairment). Possibly, this relates to the changing personal needs of PMI in these key moments. This was also described by Peek et al. [39, 40] who reported that challenges related to independent living can cause older adults to be more attracted to a technological solution or, on the contrary, to decrease or reject the use of a pre-owned technology that became less useful or too difficult to handle. We identified needs that are associated to cognitive decline (e.g. memory training and support and alleviating the ICG burden) as well as needs that occur among the broader older adult population (e.g. safety, social contact, leisure and fun, etc.). Our results show that introduced technologies need to meet at least one of these personally relevant needs and thereby needs to be perceived as useful to have a chance of getting adopted by the PMI. This finding is in line with previous work [34, 37, 49] and suggests that successful technology adoption is primarily need-driven and strongly related to the classical concept of perceived usefulness [30].

Third, we identified determinants for technology adoption and continued use in PMI and were able to distinguish three types: individual, technological and contextual factors. Thereby, different types of factors could refer to the same underlying factor (e.g. financial resources, cost and reimbursement referred to affordability of technologies), however, this classification allowed us to break down these larger factors and gain insight into the different levels of these factors. Although this classification resulted from the inductive data-analysis and was not based on a predefined theoretical framework, it can be referred back to previous work of Thordardottir et al. [36] in which similar categories were identified. Most determinants identified in our study are in line with previous research on technology acceptance in older adults with [34, 36, 37] and without [34, 39, 49, 50] cognitive impairment. Among others, these included personal attitude and interest of PMI and their environment, as well PMI’s beliefs about the impact of adoption on their sense of autonomy. The latter is closely related to the sense of control older adults perceive when interacting with technology, contributing to their satisfaction and well-being [37]. The possibility to customize (e.g. the difficulty level of a game) a technology to the PMI’s preferences, needs or ability level is related to this factor, as technologies that don’t allow this customization were criticized for ignoring the PMI’s residual functional and cognitive capacity. The desire to preserve the sense of control in PMI goes even further, as ICG and PCG stated that voluntariness of the PMI in the adoption process as well as control over privacy were important. Thereby, ICG and PCG show that technology adoption by PMI should contribute to their empowerment. Efficacy beliefs (i.e. subjective proficiency) and digital literacy level were also identified as determinants and are recurring factors in literature [39, 40]. Other major determinants were familiarity and compatibility of a technology with the PMI’s lifestyle and the possibility to integrate the use of the technology into the PMI’s routine, which were also previously identified in research [36, 40, 41]. The identification of accessibility factors (i.e. for physically/cognitively impaired or those without internet connection) and ease of use also confirmed earlier research findings [34, 36, 49]. As expected from previous research [34, 37, 39, 40, 49, 50], social support was highly important for adoption and use in PMI. Social networks can motivate and encourage PMI to adopt new technologies, advise them in their search for technologies and assist them in learning or using technologies. In contrast, social contacts can also obstruct technology adoption if they serve as an alternative to technology, delivering care or fulfilling the needs of the PMI. This was also considered by Peek et al. [39, 49], who described that technology is only one of several behavioural options older adults use to cope with challenges or increasing needs. Furthermore, technological factors were identified, such as design, reliability and interoperability. Although most of these seem important for technology acceptance in older adults, they have not been widely studied [35, 51]. Affordability was related to the concept of price value [52], as PCG’s expectations of technological reliability increased with increasing costs. According to PMI and ICG, policy measures (i.e. reimbursement) could help improve this determinant of adoption. However, perhaps even more important is the lack of knowledge of the market supply of technologies for PMI impeding the successful recommendation of technology by ICG and PCG and ultimately the implementation of technologies by PMI.

Fourth, although our data conform with the earlier discussed models of technology acceptance, we identified aspects to technology adoption and use specific to the population of PMI that have not been extensively studied and can therefore be considered novel contributions to the field. First, we identified specific needs in PMI that were related to cognitive decline. These included the need for memory training to prevent or delay further deterioration and support of daily activities (e.g. medication management) to meet the need for autonomy. Second, in PMI, the influence of technological attitudes on adoption is mediated by disease awareness. As PMI sometimes lack awareness of cognitive impairment [53] and subsequently don’t experience additional needs, they tend to ignore or reject the need for, and therefore adoption of, technological solutions. In contrast, the awareness of cognitive impairment and decreased functional ability can facilitate the technology adoption process. Therefore, disease awareness in PMI is an essential factor for adoption. Lastly, cognitive impairment is a specific barrier to sustained technology use in PMI, as technology can be forgotten to be used or the ability to use can be lost. Integration of technologies in the daily routine of PMI and social support can serve as a buffer to this and help preserving their continued use.

### Strenghts and limitations

To the best of our knowledge, this study is the first qualitative study that included technology-using and non-using PMI, additional perspectives (ICG and PCG) and different data-collection methods to inductively examine the determinants of technology adoption and continued use in PMI. The inductive data-analysis permitted the unprejudiced identification of themes from the data, as opposed to the limited previous work that made use of predefined theoretical frameworks to analyse their data [34, 37]. Moreover, the purposive sampling technique, resulting in the inclusion of technology-using and non-using PMI, differs from previous work in which mainly digitally literate PMI were included [37]. This permitted to get a clear understanding of barriers to technology adoption in the population of PMI, in addition to facilitators. Moreover, the differentiation of data related to adoption and continued use facilitated interpretation of the dynamics of determinants across implementation stages. Although this is important for gaining a good understanding of technology acceptance [40, 41, 54], this is often overlooked in previous research on this topic in PMI [34, 37]. Therefore, this study adds to the limited evidence base of studies exploring the specific determinants of technology adoption and continued use in PMI. Lastly, all data were collected by an experienced gerontopsychologist and almost all interviews took place in the home environment of the PMI. These measures potentially optimized the cooperation of participants and, potentially, the methodological quality of this study.

However, our study findings should be interpreted in the context of a few limitations. First, the results from our study are only limitedly transferable, as we recruited only in the Flanders region of Belgium. Second, although it is a strength that different perspectives were included, data-saturation from the perspective of the PCG was possibly not reached as we only conducted one focus group. Third, nearly all data were collected before the onset of the COVID-19 pandemic. This might influence the transferability of the research findings to the mid- and postpandemic context. However, the findings resulting from this study are comparable to those of Haase et al. [50] that were collected in the general older adult population during the pandemic. It is thus likely that the pandemic mainly impacts the need for technological solutions, but that the mechanisms for technology acceptance in PMI remain the same.

### Future directions

As the research base on technology acceptance in older adults is already extensive, we would like to point out opportunities for future research in this field, specifically targeted towards the population of PMI. First, additional qualitative research in PMI examining and validating our study’s findings can contribute to the development of a novel theoretical model of technology acceptance in PMI. Moreover, repetition of this study post-COVID-19 pandemic could be interesting to verify whether the current model of technology acceptance among PMI withstands the changing times. Second, previous research identified a range of technological solutions that have proven effective for supporting PMI (Dequanter et al., 2021). However, as our study results point out the barriers and facilitators to actual adoption and use in this population, future research should focus on weighting determinants and identifying those that are crucial for use behaviour. Also, future research should focus on identifying or further developing strategies to remove barriers for effective adoption and continued use in this population. We also propose some recommendations for clinical practice. As disease awareness appears to be an essential factor for technology adoption in PMI, measures to improve this should be taken in the early stages of neurocognitive decline. Moreover, as adoption is need-driven and subject to individual, technological and contextual characteristics, technology developers should work from the onset of a project with PMI and ICG, to make sure the developed technologies correspond to their individual needs and preferences. Since continued use of technology in PMI is sensitive to disruption by decreased or lost proficiency to use, strategies to increase (social) support and integration of use behaviour in daily routines of PMI are also important. Lastly, our study’s findings offer a few policy recommendations. As PCG have limited knowledge of existing technologies and resources, and are therefore limited to integrate them in clinical practice, further efforts should be made to adequately train and support them. Moreover, development of decision support systems helping PMI, ICG and PCG to select the technologies best suited to the PMI’s needs is needed. Finally, as affordability seems an important factor for adoption, more efforts should be invested into the further development and implementation of national reimbursement systems for medical health devices and health applications.

## Conclusions

The findings of this qualitative study contribute to the limited body of evidence concerning technology adoption and continued use among community-dwelling cognitively impaired older adults. They underline that successful technology adoption is primarily need-driven and subject to individual, technological and contextual characteristics. Specific for this population are the importance of disease awareness and cognitive ability for adoption and continued use. Although social support can be a valuable alternative to technology, it is an important facilitator of continued technology use in these older adults. Similarly the integration of technologies in the daily routine of cognitively impaired older adults can buffer the discontinuation of technologies. Future research in this field is encouraged to validate the findings of this study in a postpandemic era and to further develop a novel theoretical framework for technology acceptance in this specific population. Moreover, identification of crucial determinants and strategies to remove barriers to adoption and continued use should be the focus of further research. Improving disease awareness ought to be the focus of clinical strategies to enhance technology adoption in PMI. Policies are recommended to invest in training and support of professional caregivers as well as in reimbursement strategies to facilitate implementation of technology in practice.

## Supplementary Information

**Additional file 1. **

## Data Availability

The datasets used and/or analysed during the current study are available from the corresponding author on reasonable request.

## Abbreviations

PMI: Persons with mild cognitive impairments
ICG: Informal caregivers
PCG: Professional caregivers
MCI: Mild Cognitive Impairment
GPS: Global Positioning System
TV: Television
COVID-19: Coronavirus disease of 2019
