# Supplementary material for: Determinants of technology adoption and continued use among cognitively impaired older adults: a qualitative study
Source: BMC Geriatr. 2022 Apr 28;22:376. doi: 10.1186/s12877-022-03048-w (PMC9047390; doi:10.1186/s12877-022-03048-w)
Supplement: Supplementary file 1 — Additional file 1. [file 12877_2022_3048_MOESM1_ESM.docx]

# Supplementary Information: interview and focus group guide

## Interview guide

*Introduction: explaining the research goal + signing the informed consent*

1. **Situating daily activities, interests and experienced difficulties**
   1. What activities do you like to do?
   2. Which of these are more difficult than back in the days? Why? (e.g. ADL, communication, orientation, remembering appointments,…)
2. **Bridge to technology perceptions/attitudes and experience**
   1. What does technology mean to you?
   2. What kind of technologies do you use in your daily life?
   3. Are there certain technologies that support you with the earlier discussed difficulties you encounter?
      1. *Show examples of technology solutions on photo-elicitation cards*
         1. What benefits could these technologies offer you? And disadvantages?
3. **Motivators and barriers for the use of technology**
   1. Which solutions would you prefer? Why?
   2. Do you think you might want to use such technology in the future? Why?
   3. What or who would influence your decision to use or not use it? (e.g. appearance, ease of use, battery life, available support, price, reimbursement options,…)

*Do you have any comments or suggestions?
Thank you for your participation!*

## Focus group guide

*Introduction: introductory round + explaining the research goal + signing the informed consent*

1. **Introduction – Ice breaker**
   1. With which eHealth technologies for older adults with cognitive impairments or informal caregivers did you already come in contact with?
2. **Practice**
   1. What are your experiences with the use of eHealth technologies in older adults with cognitive impairments or their informal caregivers?
   2. How could these eHealth technologies support older adults with cognitive impairments? (i.e. utility/usefulness/added value)
   3. Are there other applications or devices that don’t exist yet, that could be interesting to support this population?
3. **Implementation**
   1. Which factors determine whether an older adult with cognitive impairment will use an eHealth technology? (facilitators/barriers)
      1. *Write them down on these post-its (time: 2 minutes) and afterwards we will sort them on the whiteboard in the categories ‘facilitators’ and ‘barriers’.*
   2. Wat could be your role, as care professionals, in the adoption and use of eHealth technologies among these older adults?
      1. Are eHealth technologies sufficiently available, known and accessible to you as care professionals?
      2. What are the sources you use to get to these eHealth technologies?
4. **Future perspective**
   1. What will be needed in the future to implement and deploy eHealth technologies among a wider audience?

*Do you have any comments or suggestions?
Thank you for your participation!*
